# Effect of Educational intervention based on Health Belief Model on promoting preventive behaviours of urinary tract infections in mothers with children under 6-Years of age

**DOI:** 10.1186/s12905-022-01981-x

**Published:** 2022-10-05

**Authors:** Zahra Bazargani, Fatemeh Sarikhani, Sadegh Karami Darenjani, Mehdi Amirkhani, Pooyan Afzali Harsini, Ali Khani Jeihooni

**Affiliations:** 1 Department of Pediatrics, School of Medicine, Fasa University of Medical Sciences, Fasa, Iran; 2 Department of Public Health, School of Health, Fasa University of Medical Sciences, Fasa, Iran; 3 Department of Public Health, School of Health, Shiraz University of Medical Sciences, Shiraz, Iran; 4 Department of Nursing, School of Nursing, Fasa University of Medical Sciences, Shiraz, Iran; 5 Department of Public Health, School of Health, Kermanshah University of Medical Sciences, Kermanshah, Iran; 6 Nutrition Research Center, Department of Public Health, School of Health, Shiraz University of Medical Sciences, Shiraz, Iran

**Keywords:** Health Belief Model, Mothers, Prevention, Children, Urinary tract infection

## Abstract

**Background:**

Children are one of the most vulnerable social groups to infectious diseases, and prevention of urinary tract infections in children is very important; therefore, the present study aimed to investigate the effect of education based on health belief model (HBM) on promoting preventive behaviours of urinary tract infection in mothers with children under 6-years of age.

**Methods:**

This quasi-experimental study was conducted on 150 women with children under 6 years of age referred to health centers in Fasa city, Iran in 2021. Subjects were selected using simple sampling method and were randomly divided into intervention (n = 75) and control (n = 75) groups. The educational intervention for the experimental group consisted of 6 virtual training sessions of 40–50 min using lecture, question and answer, group discussion and video clips. Two virtual follow-up sessions were also held one month and two months after the educational intervention. Three months after the educational intervention, both experimental and control groups completed the questionnaire. Data were analysed by using SPSS 22 through Chi-square, independent t-test, and paired t-test (p > 0.05).

**Results:**

Before the intervention, based on independent t-test and paired t-test, the mean score of HBM constructs were not significantly different between the control and intervention groups (P > 0.05). However, while perceived barriers significantly decreased (P < 0.05) after the intervention, the mean score of knowledge, ‌ perceived sensitivity and severity, perceived benefits, ‌ self-efficacy, cues to action, and performance significantly increased (P < 0.05) after the intervention.

**Conclusion:**

Considering the effect of training preventive behaviours of urinary tract infection based on HBM, application of the model as an effective and cost-effective method along with other methods is recommended for educational programs of mothers with children under 6 years of age.

## Background

Urinary tract infection is the most common disease of the urogenital tract and the second most common bacterial disease in children. Numerous factors are involved in the development of urinary tract infections including female gender, functional disorders of urinary excretion, obstructive uropathy, constipation, and vesicoureteral reflux[1]. Accompanying symptoms in children with urinary tract infections include pain in the sides and abdomen, moodiness, urinary burning, recurrent urination, growth retardation, diarrhea, fever, blood in the urine, anorexia and nutritional problems, urinary incontinence, poor urine flow or urinary dribbling, restlessness, genital burning, constipation, pyuria, nocturia, vomiting, and fever unexplained. The site of involvement is mainly in the ureters, bladder and urethra, and most symptoms are local though clinical manifestations do not always indicate the exact location of the infection[2].

Being asymptomatic and difficulty in getting a urine sample, urinary tract infections in children are a challenging behavior in primary care. On the other hand, children are the most vulnerable social groups to infectious diseases. The high prevalence of infection, the possibility of recurrence of the disease, the variety of clinical manifestations at different ages, the subsequent difficulty of clinical and laboratory diagnosis, resistance of the disease agent to antibiotics, and long-term serious complications make urinary tract infections especially important in children[3]. In many cases, the disease can be prevented by appropriate health behaviors leading to minimal risk. Although today, with the help of modern diagnostic and therapeutic methods, mortality from the disease is approaching zero, urinary tract infection is an important factor in causing progressive damage, renal failure, urinary stones, and hypertension in children[4].

One of the most important and basic tasks of the health care system in the face of urinary tract infections is the proper identification and treatment of the patients because if not diagnosed and treated in time, children will be prone to irreversible complications[5] .

Urinary tract infections (UTIs) are common bacterial infections in children, affecting around 1·7% of boys and 8·4% of girls before the age of 7 years and During the first year of life UTIs affect boys and girls equally, but after that age most cases occur in girls[6]. Up to 8% of children will experience at least one UTI between the ages of 1 month and 11 years. In the United States, there are about 1.5 million pediatric ambulatory visits annually for UTIs[7].

The effect of health education programs depends to a large extent on the correct use of related theories and models. Behaviors, health manners, adhering to these actions while being aware of the predisposing factors of urinary tract infections and by making changes in these behaviors based on targeted intervention has an effective role in reducing and preventing urinary tract infections, especially in children[8].

Knowledge does not merely lead to behavior change, it is a need to change behavior[9]. One of the conceptual frameworks used to develop the training program is the HBM, which studies behavior in health education. Key constructs of this model include perceived sensitivity (one’s sensitivity to a particular disease), perceived severity (one’s beliefs about the severity of the disease), perceived benefits (one’s perception of the benefits of adopting preventive behavior), perceived barriers (one’s perception of the obstacles blocking the way of any health behavior), cues to action (stimuli that accelerate making a decision and urge to perform a behavior), and self-efficacy (one’s strong belief in his or her ability to successfully perform a behavior)[10]. In a quasi-experimental study by Hashemiparast et al., educational intervention based on HBM had a positive effect on urinary tract infection prevention behaviors in mothers. The health belief model improved the mean score of perceived benefits, perceived severity, and mothers’ self-efficacy[11]. In a study by Mousavi et al., training reduced recurrence of urinary tract infections in children and the mean score of HBM constructs increased in both groups of intervention and control[12].

Most of the studies conducted in Iran regarding pediatric urinary tract infections were solely limited to descriptive studies related to diagnostic and therapeutic methods, and few investigated the prevention methods. Due to the relatively high prevalence of urinary tract infections in children, their acute and chronic complications, their heavy economic burden on families and society, and prominent role of health behaviors in preventing these infections, various interventions are highly recommended to increase behaviors preventing the infections. Educational programs solely aimed to increase awareness often succeed in this goal, but are usually not effective in changing other variables such as perceptions and beliefs and creating sustainable behaviors[12]. Accordingly, due to the importance of prevention and timely diagnosis of the disease in preventing its secondary complications, the present study aimed to investigate the effect of education based on health belief model (HBM) on promoting preventive behaviours of urinary tract infection in mothers with children under 6-years of age.

## Methods

This quasi-experimental study was conducted on 150 women with children under 6 years of age referred to health centers in Fasa in 2021. First, from among all health centers in Fasa city; Iran (6 centers), 2 centers were selected using randomly. Then, participants were recruited using a sampling method from each center(75 mothers in each group) and then were randomly divided into intervention (n = 75) and control (n = 75) groups according to their household number registered in their health file. After stating the objectives of the study, informed consent was obtained from participants. The formula for calculating the sample size is as follows:


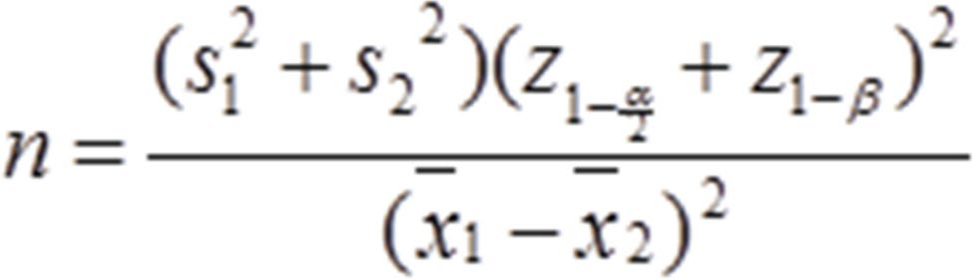


## Inclusion and exclusion criteria

Inclusion criteria were mothers with children under 6 years of age, willingness to participate in educational programs, literate. Exclusion criteria were absence in more than one training session, unwillingness to participate in the study, and failure to complete questionnaire.

**Fig. 1:**
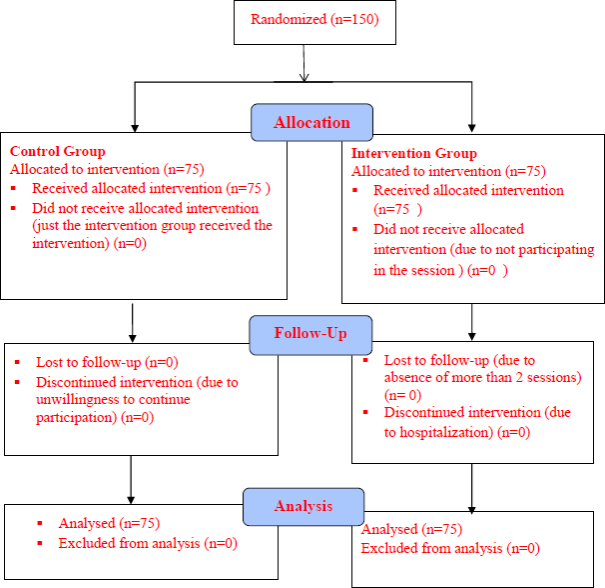
Flow Chart of the Study

After selecting the samples that met the inclusion criteria, they were randomly divided into experimental and control groups. Figure 1 presents the study flow chart. Data collection tools included demographic characteristics, knowledge assessment, an HBM-based questionnaire, and performance assessment[11]. Mothers’ knowledge about urinary tract infections was measured with 20 multiple-choice questions, scoring zero to 20.

## Educational intervention

The data collection tool was a questionnaire designed based on the health belief model. The first part questions on the HBM constructs includes questions related to measuring the demographic characteristics such as mother’s occupation, father’s occupation, monthly household income, mother’s education, father’s education and children’s gender.

The questions on the HBM constructs were based on a 5-point Likert scale (‘strongly disagree’ to ‘strongly agree’), scoring 1 to 5. Knowledge was measured by 20 questions in which the “yes” answer had 1 score and the “no” or “no idea” answer had a zero score (scores of 0 to 20). The perceived sensitivity, severity, advantages, and self-efficacy were examined using five questions with point values ranging from 5 to 25. While cues to action were tested with three questions yielding scores between three and fifteen, perceived barriers were measured with seven questions yielding scores between seven and thirty-five. The performance was also measured by four 5-point Likert scale questions (ranging from “I’m not quite sure” to “I’m totally sure”), scoring between 4 and 20. In a study by Hashemiparast et al., the validity and dependability of the instruments were established. Cronbach’s alpha was 0.88 overall. Cronbach’s alpha was also 0.89 for knowledge, 0.82 for perceived susceptibility, 0.84 for perceived severity, 0.80 for perceived advantages, 0.82 for perceived barriers, 0.80 for self-efficacy, and 0.82 for Cues to action. [11].

The goal of the study and the procedures for completing the tasks were explained to participants and health center workers. The questionnaire was filled out by experimental and control groups.Based on the results of the pre-test, the educational content was developed using the HBM. The experimental group engaged in six 40- to 50-minute virtual training sessions that included lectures, questions, group discussions, instructional visuals, video clips, and PowerPoint presentations. A weekly instructive text message is sent to the experimental group in order to retain and encourage their participation. In addition, a WhatsApp group was established for information exchange. Two virtual follow-up meetings were held one month and two months after the educational intervention. Both the experimental and control groups completed the questionnaire three months after the educational intervention. At the completion of the study, the entire instructional material was supplied to the control group as a virtual educational package. (Table 1).

**Table 1:** Description of training sessions held in the experimental group

Number of sessions	The educational content
**1**	Familiarity with urinary tract infection in children, high prevalence of urinary tract infection, risk factors, the possibility of recurrence of the disease, predisposing factors for recurrent urinary tract infection, variety of clinical manifestations at different ages (in order to increase the structure of knowledge and perceived sensitivity)
. **2**	The training program was conducted by a Ph.D. of health education and health promotion and a medical student in collaboration with a nephrologist. Many different topics were discussed during the sessions, including subsequent difficulty of clinical diagnosis, resistance of the causative agent to antibiotics, long-term serious complications in children, proper identification, treatment methods, the most important preventive measures, the benefits and barriers to preventive behaviors, and role of mothers. (in order to increase the structure of perceived sensitivity)
. **3**	The mother of an 7-year-old child who had a urinary tract infection was invited and she spoke to people about the urinary tract infection and the problems caused by it. In this meeting, it was emphasized to change the positive attitude of people to prevent urinary tract infection and the role of mothers in proper activities (in order to increase the structure of perceived severity)
. **4**	mention to the role of proper identification, treatment methods, the most important preventive measures, the benefits and barriers to preventive behaviors, and role of mothers. (in order to increase the structure of perceived benefits).
. **5**	Each woman was also asked to discuss her prior experiencesAlso, the mothers discussed with each other about the Perceived barriers and the strategies to deal with it (to reduce the Perceived barriers).
**6**	During a training session, people were taught about the most important preventive measures with the help of each other, They performed a number of appropriate actions in a demonstration (in order to increase self-efficacy structure).At the end of the sessions, the entire educational content was sent to participants as a virtual training package Also during training sessions, educational images, video clips, and PowerPointwas provided to the participants of the intervention group (Cues to action)

## Ethical considerations

While obtaining permission from the ethics committee of Fasa University of Medical Sciences (design code: 97,479- IR.FUMS.REC.1400.076 ethics code) and Fasa Health Centre, and obtaining informed consent from subjects, participants were assured that their information would remain confidential.

### Data analysis

Data were analysed by using SPSS 22 through Chi-square, independent t-test, and paired t-test (p>0.05).

Based on the results of the independent *t-*test, as shown in Table 2, the mean scores of knowledge, perceived sensitivity, perceived severity, perceived benefits, perceived barriers, self-efficacy, cues to action, and performance of the experimental and control groups before intervention were not significantly different between the two groups (P > 0.05) that showed all data was homogeneous.

**Table 2:** Comparison of mean score of Knowledge and Structures of HBM model of the experimental and control groups before and three months after the intervention

Variables	Group		Before the intervention	3 months after the intervention	p-value
Knowledge	experimental	9.24±1.58		16.1±15.55	0.001
	control	10.1±17.14		11.02±1.10	0.259
	p-value	0.248		0.001	
Perceived sensitivity	experimental	10.1±23.38		20.1±78.41	0.001
	control	10.1±56.82		11.1±12.70	0.278
	p-value	0.293		0.001	
Perceived severity	experimental	9.1±14.68		19.1±79.52	0.001
	control	10.22±1.08		11.1±47.02	0.284
	p-value	0.266		0.001	
Perceived benefits	experimental	12.1±68.66		21.10±29.48	0.001
	control	13.1±5.42		13.1±95.40	0.246
	p-value	0.272		0.001	
Perceived barriers	experimental	26.67±2.33		10.17±2.29	0.001
	control	25.2±94.27		25.2±02.30	0.291
	p-value	0.208		0.001	
Perceived self-efficacy	experimental	10.14±1.40		21.16±1.59	0.001
	control	10.1±66.72		11.1±74.70	0.251
	p-value	0.287		0.001	
Cues to action	experimental	7.10±0.84		12.0±75.72	0.001
	control	7.0 ±58.76		7.0±84.72	0.285
	p-value	0.258		0.001	
Performance	experimental	8.78±1.14		17.1±09.20	0.001
	control	8.1±13.36		8.1±74.40	0.279
	p-value	0.288		0.001	

## Results

In this study, 150 women with children under 6 years of age participated. The mean age of mothers in the experimental and control groups was 36.28 ± 4.70 and 35.84 ± 4.27 years, respectively (p = 0.144). The mean age of children in the experimental and control groups was 45.13 ± 69.23 and 47.12 ± 30.81 months, respectively (p = 0.126). The mean family dimension of the experimental and control groups was 3.84 ± 1.16 and 3.95 ± 1.06, respectively (p = 0.194). Based on the t-test, there was no significant difference between the two groups. The history of urinary tract infection among the children in both groups was 18%.

Based on the Chi-square test, there was no statistically significant difference between the two groups in terms of mother’s occupation (p = 0.125), father’s occupation (p = 0.134), monthly household income (p = 0.107), mother’s education (p = 0.122), father’s education (p = 0.159) and children gender (p = 0.210) (Table 3).

**Table 3:** Comparison of frequency distribution of demographic characteristics between experimental and control groups

variables		Experimental group		Control group		P-value
		number	percentage	number	percentage	
Mother’s occupation	Housewife	57	76	52	69.33	0.125
	Employed	18	24	23	30.67	
Father’s occupation	Employed	18	24	20	26.67	0.134
	Worker	10	13.33	13	17.33	
	Self-employed	27	36	24	32	
	Other	20	26.67	18	24	
Monthly household income	<40 million Rials	28	37.33	24	32	0.107
	40-80 million Rials	32	42.67	35	46.67	
	˃80 million Rials	15	20	16	21.33	
Mother’s education	Primary school	6	8	8	10.67	0.122
	Secondary school	18	24	16	21.33	
	High school	32	42.67	36	48	
	College	19	25.33	15	20	
Father’s education	Primary school	5	6.67	7	9.33	0.159
	Secondary school	12	16	18	24	
	High school	40	53.33	36	48	
	College	18	24	14	18.67	
Children’s gender	male	36	48	37	49.33	0.210
	female	39	52	38	50.67	

The results showed that before the educational intervention, there was no significant difference between the experimental and control groups in terms of knowledge, perceived sensitivity, perceived severity, perceived benefits, perceived barriers, self-efficacy, cues to action, and performance; however, three months after the educational intervention, the experimental group showed a significant increase in the mentioned variables except perceived barriers (Table 2).

## Discussion

Based on the results of the study, knowledge of the subjects in the intervention and control groups was not significantly different before the intervention, indicating the homogeneity of the study samples; however, after the intervention, knowledge of the subjects in the experimental group significantly increased, indicating the effect of educational intervention, which was consistent with the results of similar studies[12–15].

The results of studies by hashemi parast et al.[11] on Design and evaluation of educational interventions on the health belief model to promote preventive behaviors of urinary tract infection in mothers with children less than 6 years show a significant difference in the level of knowledge of the participants in the experimental group were consistent with the results of our study. The results of studies by Sadeghi et al.[16], Javaheri et al.[17] and rahimi et al.[18] were consistent with the results of our study regarding effect of an HBM-based intervention on promoting knowledge and performance of mothers with children under 6 years of age.

The more mothers are aware of urinary tract infection in children, high prevalence of urinary tract infection, risk factors, the possibility of recurrence of the disease, predisposing factors for recurrent urinary tract infection, variety of clinical manifestations at different ages, subsequent difficulty of clinical diagnosis, resistance of the causative agent to antibiotics, long-term serious complications in children, proper identification, treatment methods, the most important preventive measures, the benefits and barriers to preventive behaviors, and role of mothers. This increased awareness is possible with education which plays an important role in prevention of children urinary tract infection.

Based on the assessment made three months after the intervention, perceived sensitivity of the experimental group significantly increased compared to control group, indicating the effectiveness of intervention in improving perceived sensitivity of the experimental group, which was consistent with the results of similar studies[12, 19].

Perceived severity of the experimental group significantly increased three months after the intervention compared to control group, which was consistent with the results of studies by Shamsi et al.[20] and Tavakoli et al.[21]. The perceived severity of growth disorders in children refers to the fact that mothers are well aware of the severe consequences of urinary tract infection and they know that urinary tract infection, may have long-term serious complications in children. also Mothers had a new understanding of their children’s vulnerability despite urinary tract infections, and this led the experimental group to take risk-reducing behaviors more seriously[22].

Perceived benefits of the experimental group significantly increased three months after the intervention compared to control group, which was consistent with the results of similar studies[17, 23–25]. Accordingly, a significant increase in the perceived benefits showed that the person had chosen the behavior with the most benefits, leading to an increase in self-efficacy. The results of a clinical trial study by Roozbehani et al. showed the significant increase of HBM constructs in the intervention group three months after the intervention[26]. The more mothers are aware of the benefits to preventive behaviors, and role of mothers. This increased Perceived benefits. that is possible with education which plays an important role in increased Perceived benefits.

The most powerful construct in describing and predicting health-protective behaviors is the perceived barriers. The results of this study showed the decrease of perceived barriers in the experimental group after the intervention. The results of several studies, including Sadeghi[19], Seyed Rajabizadeh et al.[14] were also consistent with the results of our study. The results of a study by Ayaz-Alkaya[27] showed the effectiveness of the HBM-based education program in motivating students to change their lifestyle, identifying perceived barriers and benefits in combating premenstrual syndrome, which were consistent with the results of our study. The results of a study by Khorramabadi et al.[28] also showed that educational interventions based on health promotion models can be effective in increasing awareness, better understanding of risks, reducing barriers to healthy behavior and ultimately improving women’s health and nutritional performance during pregnancy. The results of a study by Zeigheimat et al.[29] showed that HBM-based education can increase nurses’ knowledge, perceived threat, and perceived benefits. The more mothers are aware of the barriers to preventive behaviors, and role of mothers. This decreased Perceived barriers. that is possible with education which plays an important role in decreased Perceived barriers.In addition, it can reduce perceived barriers and improve control of nosocomial infections among nurses. HBM is also a valuable framework for planning intervention programs.

The most powerful construct in preventing behavior change is self-efficacy. People with maximum behavior change have higher self-efficacy to perform behavior. The results of a study by Rahimi et al.[18] showed that self-efficacy has the greatest effect on behavior. In this study, the mean score of self-efficacy in the experimental group significantly increased after the intervention, indicating the effectiveness of intervention, which is consistent with the results of a study by Sang et al.[30] and other studies[12, 14, 31].

In terms of cues to action and performance, the results showed no significant difference between the experimental and control groups before the intervention; however, three months after the intervention, both variables had a significant increase in the experimental group. The results of several studies, including Sadeghi et al.[16], Javaheri et al.[17] and Rahimi et al. [18] were also consistent with the results of our study, indicating the effectiveness of the intervention in choosing proper cue to action by mother.

## Strengths and limitations

One of the strengths of the present study was the use of web-based training as a complementary training method, which, given the current state of COVID-19 pandemic, it was an effective step towards promoting and developing new education and preventing further outbreaks of the disease. Another strength of the present study was the use of focus group method, videoclips, and experienced professors to teach the relevant content.

The present study was limited to mothers with children under 6 years of age, so generalization of the results to other population should be avoided. Other limitations of the present study included the heterogeneity of participants’ education, the absence of some participants in some training sessions, the use of self-report to collect information, and communication disorders in web-based sessions. Also, when referring to the health centers, it was possible to participants to exchange information.

It is suggested that the present study be performed in a longer follow-up period, including 6 months and one year, and in different clinical settings (including hospitals, clinics and offices) in the future. The study can also be performed to evaluate preventive behaviors of other infectious diseases and with participants of different ages.

## Conclusion

Promoting mothers’ knowledge of preventive behaviors of urinary tract infections in children and their understanding of the risks and complications of these infections through appropriate educational models such as HBM in educational-therapeutic environments leads to a significant reduction in disability and long-term complications of the infections as well as the cost of hospitalization and treatment. In this study, the HBM-based educational intervention could improve the preventive behaviours of urinary tract infection; therefore, our suggestion is that the Theory could be used as a suitable framework in a wider population to improve the preventive behaviours of urinary tract infection.

## Data Availability

The datasets used and/or analysed during the current study are public data available from the corresponding author request.

## Abbreviations

HBM: Health Belief Model.
